# Compared With Girls, Boys' Psychological Symptoms Are More Likely to Be Influenced by Lifestyle in Chinese Middle School Students

**DOI:** 10.3389/fpsyg.2022.899273

**Published:** 2022-07-08

**Authors:** Jinkui Lu, Chun Tan, Jianfeng Zhou, Mian Sha, Yongli Xu, Jianhua Qiu, Ningling Liu

**Affiliations:** ^1^School of Physical Education, Shangrao Normal University, Shangrao, China; ^2^Department of Preschool Education, Shangrao Preschool Education College, Shangrao, China

**Keywords:** lifestyles, middle school students, psychological symptoms, correlation analysis, gender differences

## Abstract

**Purpose:**

To explore the relationship between Chinese middle school students' lifestyles and psychological symptoms and compare the differences between genders.

**Method:**

Using the method of stratified cluster sampling, 14,356 students aged 13–18 years from 8 cities in China were selected as participants. The Multidimensional Sub-health Questionnaire of Adolescents was used to estimate psychological symptoms. Chi-square test and Logistic regression analysis were used to analyze the relationship between lifestyle and psychological symptoms.

**Results:**

The psychological symptom detection rate of Chinese middle school students was 21.37%. The detection rate of psychological symptoms for boys was 22.1%, which was significantly higher than for girls (20.64%, χ^2^ = 4.608, *P* < 0.05). According to the Logistic regression analysis, factors that have a positive correlation with the demonstration of psychological symptoms (*P* < 0.01) include: how the students go to school (by foot or on the vehicle) (OR = 1.16, 95% CI: 1.07–1.25); inadequate time for sleep (OR = 1.48, 95% CI: 1.28–1.72); video watching time ≥2 h/d (OR = 1.25, 95% CI: 1.13–1.39); never exercise (OR = 2.39, 95% CI: 2.07–2.76); never participate in extracurricular exercises (OR = 1.45, 95% CI: 1.27–1.66); have breakfast occasionally (OR = 1.35, 95% CI:1.22–1.50); never have breakfast (OR = 1.90, 95% CI: 1.62–2.24); always have snacks (OR = 1.27, 95% CI: 1.13–1.44); always drink sugared beverages (OR = 1.37, 95% CI: 1.22–1.55); picky with food occasionally (OR = 1.22, 95% CI: 1.11–1.33).

**Conclusions:**

There was a positive correlation between unhealthy lifestyle and the occurrence of psychological symptoms, and boys are more easily influenced by lifestyles than girls.

## Introduction

Adolescence is an unstable stage of immature mental health development (Kingsbury et al., 2020; Orben et al., 2020; Zhang et al., 2020; Willinger et al., 2021). Adolescent mental health is very important to their behavioral development and any unwanted event will impact their fragile mentality and may leave traumas on their psyche (Zimet and Jacob, 2001). According to WHO (2020), there are about 10–20% of teenagers worldwide plagued with multifarious psychological symptoms. It was reported that approximately 1 trillion dollars in the economy are lost every year in the world because of multifarious psychological symptoms (Younger, 2016). Worse still, presence of the minor psychological symptoms in adolescence are likely to cause harsh consequences including non-suicidal self-hurt, depression, anxiety, and even suicide when they become adults (Resnick et al., 1997; Kieling et al., 2011; Perou et al., 2013; Schoeps et al., 2018).

Research has confirmed that teenagers living a depraved life regarding their health have a higher risk of being diagnosed with psychological symptoms (Kim and Kim, 2017). Mozzillo et al. (2021) suggested that the health-related quality-of-life (HRQOL) will increase by 12.2% with more scores on dietary habit. Recently, the COVID-19 pandemic has forced the lifestyle of the teenagers to take a U-turn, with more time watching the video, less time on sports and exercise, eating too many snacks, and binging on sugared beverages (Nikolaidis et al., 2021, 2022). As a consequence, the physical and mental health of teenagers will be unavoidably impaired by this sick lifestyle. Research showed that eating fatty or high-calorie food and drinking too many sugared beverages will make the kid easier to contract psychological symptoms (Kuhl et al., 2012; Myde et al., 2014).

Meta-analysis suggested that Chinese adolescents' mental health deteriorated across birth cohorts since the early 1990s, shown in increased scores on the negative indicators of mental health (e.g., mental problems, anxiety, and depression) (Xin et al., 2012). Moreover, 1.2 billion dollars of loss are also caused every year because of the psychological problems of the teenagers in China (Xu et al., 2019b). To our best knowledge, we only found one study focused on the relationship between lifestyle and mental health in China (Li et al., 2021). Given the serious psychological problems of Chinese teenagers, the present study aimed to estimate the relationships between lifestyles and psychological symptoms of Chinese middle school students.

## Materials and Methods

### Data Source and Participants

In this study, PASS software was used to calculate the sample size. The sample size of this study can reflect the relationship between lifestyle and psychological symptoms (Wang and Sun, 2016). Participants were distributed in the Jilin, Heilongjiang, Anhui, Shanghai, Henan, Xinjiang, Guangzhou, and Hainan provinces of China. In each province, 4 middle and 4 high schools were selected, and 2 teaching classes in each grade in each school were randomly selected. Consequently, 384 classes were selected with 35–40 students in each class, and students in these selected classes without mental and physical diseases were recruited as participants. Finally, a total of 14,869 middle school students were recruited for the present study. After excluding 333 participants (2.27%) because of missing data or extreme values, 14,356 valid data were obtained. The survey was approved by the Ethics Committee of the School of Physical Education of Shangrao Normal University (2018R-0219). This study was carried out following the Declaration of Helsinki. All the student's names were numerically coded to avoid personal information leaking. All the participants and their parents signed written informed consent.

### Procedures

The study was conducted from April to July 2018, by trained staff which was comprised of postgraduate students who majored in human movement science and PE teachers. Before the test, the students were told the purpose, requirements, and significance of the study with consistent words for every class. After being assigned the questionnaires, the students were asked to complete them with the staff present. The questionnaires were retrieved on the spot.

### Questionnaires

The questionnaire included demographic information and questions on the lifestyles and psychological symptoms. Demographic information included age, gender, grade, region city, etc. The questionnaire was determined after a pre-survey with an interval of 15 days with a Cronbach α coefficient of 0.87.

Psychological symptoms were tested by the Multidimensional Sub-health Questionnaire of Adolescents (MSQA), which was specially designed for teenagers (Tao et al., 2008). The scale had a Cronbach α coefficient of 0.963 (Chao et al., 2008; Yu-hui et al., 2008) and has been proved to be valid for the survey on the psychological symptoms of Chinese teenagers (Cao et al., 2011; Wan et al., 2015; Wu et al., 2015; Xu et al., 2019a; Zhang et al., 2020). The scale was composed of three dimensions which were emotional symptoms, behavioral symptoms, and social adaptation difficulties with 18, 8, and 13 entries under each dimension respectively. Each entry had a scale of 1–6 and the participants can choose the number according to their behaviors. Reverse scoring was used in the statistical organization. When a certain behavior endured for 1 month or more, the student was defined as having psychological symptoms. When the student had more than three behaviors under the category of emotional symptoms and they lasted over 1 month, he was diagnosed with emotional symptoms; the same with the behavioral symptoms and social adaptation difficulties. If the student had more than one behavior that last over 1 month under the category of behavioral symptoms, then he was diagnosed with behavioral symptoms. For the diagnosis of adaptation difficulties, the threshold number was four. If the addition of these values under the three categories was larger than 8, the student was defined as having psychological symptoms.

The investigation on lifestyle included: How to go to school and come back from school? How many hours of sleep per night? How many hours of watching videos? Exercising frequency at school? How many hours of exercising at school?

Exercise after class? How regularly do they have breakfast? How often do they have snacks? How often do they drink sugared drinks? How pick they are with the food? These items were prepared and discussed by relevant experts with reference to the Chinese National Survey on Students' Constitution and Health (CNSSCH Association, 2016).

### Stats and Analysis

The chi-square test (χ^2^ test) was used to compare the detection rates of the psychological symptoms as well as each dimension of the Chinese teenagers. The correlations between lifestyles and psychological symptoms for boys and girls were analyzed using the Logistic regression method after gender, grade, and city were controlled. The Logistic regression analysis was conducted on the gender difference based on the reference of girls, with the factors of grade and city control. Epidata entry 3.1 was used for data entry and was double-checked to guarantee accuracy. SPSS25.0 software was used for data analysis and α = 0.05 was the two-sided test level.

## Results

**Figure 1:**
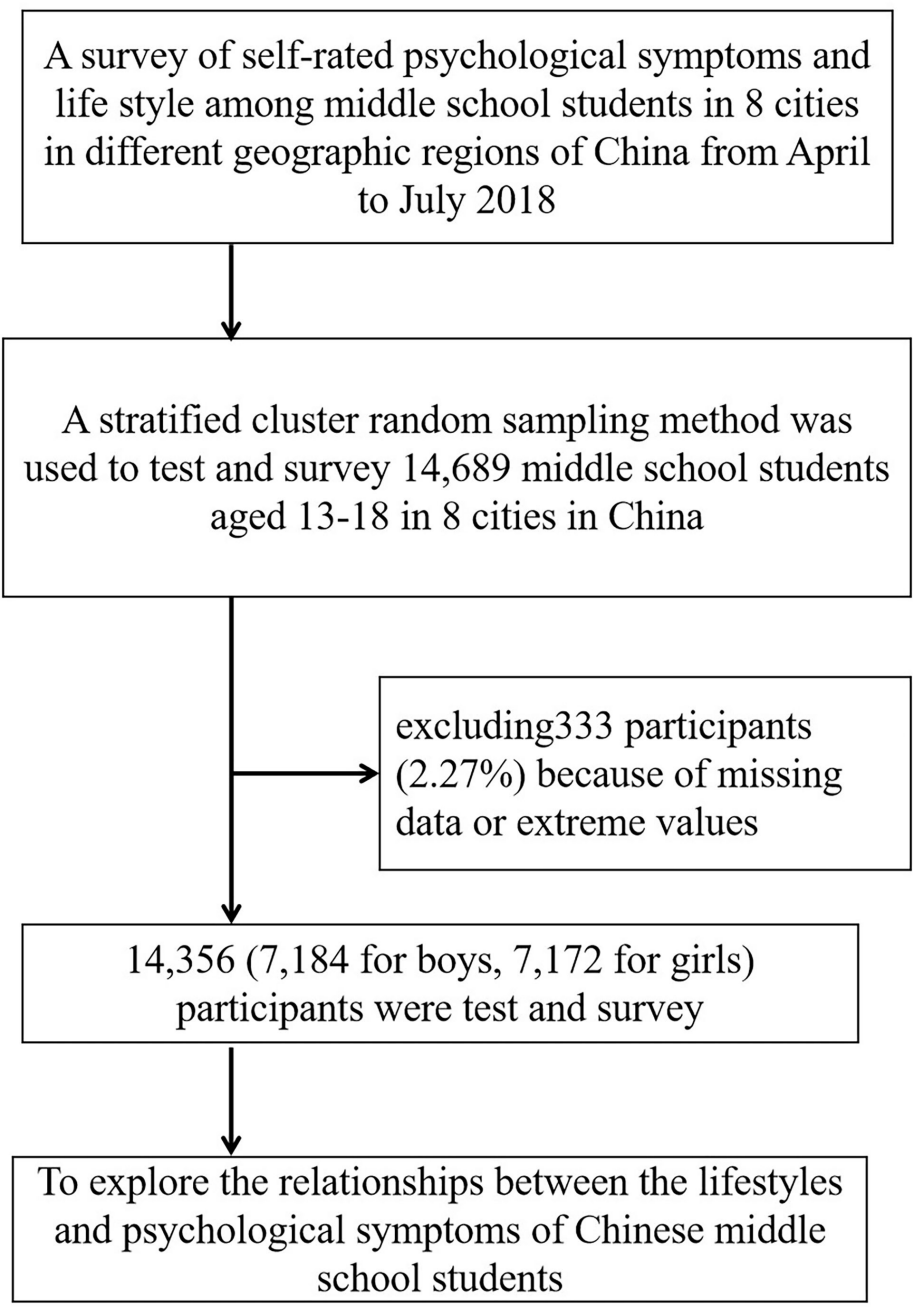
Sampling flow chart of Chinese middle school students.

A total of 14,869 middle school students (7,184 boys, 50.4%; 7,180 junior middle school students, 50.01%) participated in the present study (Figure 1). The average age of the surveyed students was (15.51 ± 1.71) years old.

Table 1 shows that the detection rate of psychological symptoms in middle school students was 21.37% (3,068/14,356). Among the three dimensions, the detection rate of emotional symptoms, behavioral symptoms, and social adaptation difficulties was 27.67% (3,972/14,356), 27.44% (3,940/14,356), and 17.11% (2,456/14,356), respectively. The detection rate of psychological symptoms in boys (22.1%) was higher than that of girls (20.64%, χ^2^ = 4.608, *P* < 0.05). The detection rate of psychological symptoms for teenagers with different lifestyles almost had statistical significance (χ^2^ = 11.359, 22.942, 21.192, 172.850, 29.202, 86.592, 45.096, 32.520, 134.994, *P* < 0.01).

**Table 1:** The comparison of detection rates of psychological symptoms with different lifestyles and for Chinese middle school students (%).

**Category**		***N***	**Emotional symptoms**			**Behavioral symptoms**			**Social adaptation difficulties**			**Psychological symptoms**		
			**Prevalence**	**χ** ^**2**^	***P*** **-value**	**Prevalence**	**χ** ^**2**^	***P*** **-value**	**Prevalence**	**χ** ^**2**^	***P*** **-value**	**Prevalenc*e***	**χ** ^**2**^	***P*** **-value**
Gender	Boys	7,184	1,996 (27.78)	0.097	0.756	2,038 (28.37)	6.161	<0.05	1,308 (18.21)	12.254	<0.01	1,588 (22.10)	4.608	<0.05
	Girls	7,172	1,976 (27.55)			1,902 (26.52)			1,148 (16.01)			1,480 (20.64)		
Grade	Junior high school	7,180	2,108 (29.36)	20.535	<0.01	2,168 (30.19)	54.553	<0.01	1,224 (17.05)	0.037	0.847	1,600 (22.28)	7.13	<0.05
	High school	7,176	1,864 (25.98)			1,772 (24.69)			1,232 (17.17)			1,468 (20.46)		
Region	City	1,0974	3,008 (27.41)	1.545	0.214	3,002 (27.36)	0.187	0.665	1,804 (16.44)	14.701	<0.01	2,280 (20.78)	9.797	<0.01
	Rural	3,382	964 (28.5)			938 (27.74)			652 (19.28)			788 (23.30)		
How to go to school and come back	Active	7,406	1,966 (26.55)	9.62	<0.01	1,982 (26.76)	3.583	0.058	1,200 (16.2)	8.83	<0.01	1,500 (20.25)	11.359	<0.01
from school	Passive	6,950	2,006 (28.86)			1,958 (28.17)			1,256 (18.07)			1,568 (22.56)		
How many hours of sleep at night	Deprivation	1,2932	3,666 (28.35)	30.159	<0.01	3,618 (27.98)	18.54	<0.01	2,286 (17.68)	29.791	<0.01	2,834 (21.91)	22.942	<0.01
	Adequate	1,424	306 (21.49)			322 (22.61)			170 (11.94)			234 (16.43)		
How many hours of watching videos	<2 h/d	1,1944	3,224 (26.99)	16.196	<0.01	3,144 (26.32)	44.953	<0.01	1,964 (16.44)	22.131	<0.01	2,468 (20.66)	21.192	<0.01
	≥2 h/d	2,412	748 (31.01)			796 (33)			492 (20.4)			600 (24.88)		
Exercising frequency at school	Never	1,364	562 (41.2)	139.951	<0.01	526 (38.56)	95.108	<0.01	382 (28.01)	128.465	<0.01	480 (35.19)	172.85	<0.01
	Occasional	9,672	2,570 (26.57)			2,514 (25.99)			1,572 (16.25)			1,952 (20.18)		
	Often	3,320	840 (25.3)			900 (27.11)			502 (15.12)			636 (19.16)		
How many hours of exercising at school	<60 min/d	12,312	3,446 (27.99)	4.455	<0.05	3,372 (27.39)	0.141	0.707	2,144 (17.41)	5.713	<0.05	2,656 (21.57)	2.091	0.148
	≥60 min/d	2,044	526 (25.73)			568 (27.79)			312 (15.26)			412 (20.16)		
Exercise after class	Yes	1,744	394 (22.59)	25.56	<0.01	434 (24.89)	6.532	<0.05	226 (12.96)	24.099	<0.01	286 (16.40)	29.202	<0.01
	No	12,612	3,578 (28.37)			3,506 (27.8)			2,230 (17.68)			2,782 (22.06)		
How regular they have breakfast	Always	11,226	2,920 (26.01)	80.774	<0.01	2,906 (25.89)	69.867	<0.01	1,778 (15.84)	77.281	<0.01	2,228 (19.85)	86.592	<0.01
	Occasional	2,386	768 (32.19)			760 (31.85)			478 (20.03)			602 (25.23)		
	Never	744	284 (38.17)			274 (36.83)			200 (26.88)			238 (31.99)		
How often they have snacks	Always	3,668	1,182 (32.22)	51.594	<0.01	1,148 (31.3)	36.856	<0.01	720 (19.63)	29.638	<0.01	922 (25.14)	45.096	<0.01
	Occasional	8,196	2,126 (25.94)			2,134 (26.04)			1,286 (15.69)			1,612 (19.67)		
	Never	2,492	664 (26.65)			658 (26.4)			450 (18.06)			534 (21.43)		
How often they drink sugared drinks	Always	2,230	750 (33.63)	46.99	<0.01	736 (33)	48.072	<0.01	478 (21.43)	35.115	<0.01	578 (25.92)	32.52	<0.01
	Occasional	7,564	2,004 (26.49)			2,062 (27.26)			1,244 (16.45)			1,556 (20.57)		
	Never	4,562	1,218 (26.7)			1,142 (25.03)			734 (16.09)			934 (20.47)		
How pick they are with the food	Never	4,308	1,038 (24.09)	125.03	<0.01	1,012 (23.49)	117.666	<0.01	648 (15.04)	83.455	<0.01	784 (18.2)	134.994	<0.01
	Occasional	8,578	2,358 (27.49)			2,368 (27.61)			1,436 (16.74)			1,806 (21.05)		
	Always	1,470	576 (39.18)			560 (38.1)			372 (25.31)			478 (32.52)		

*N, Number; χ^2^, Chi-square*.

Table 2 showed the logistic regression analysis of the correlations between lifestyles and psychological symptoms of Chinese middle school students. We found that psychological symptoms were positively correlated with (a higher OR value indicates a stronger correlation): the way to and from school passively (OR = 1.16, 95% CI: 1.07–1.25), sleeping < 8 h at night (sleep deprivation) (OR = 1.48, 95% CI: 1.28–1.72), watching TV/using the digital device more than 2 h per day (OR = 1.25, 95% CI:1.13–1.39), never exercise at school (OR = 2.39, 95% CI: 2.07–2.76), never exercise after class (OR = 1.45, 95% CI: 1.27–1.66), eating breakfast occasionally (OR = 1.35, 95% CI: 1.22–1.50), never eating breakfast (OR = 1.90, 95% CI: 1.62–2.24), always eating snacks (OR = 1.27, 95% CI: 1.13–1.44), always have sugared beverages (OR = 1.37, 95% CI: 1.22–1.55), picky with food occasionally (OR = 1.22, 95% CI: 1.11–1.33), often picky with food (OR = 2.24, 95% CI: 1.95–2.56) (*P* < 0.01).

**Table 2:** Logistic regression analysis on the correlations between lifestyles and psychological symptoms of Chinese middle school students.

**Independent variable**		**β value**	**Standard error**	**Wald χ^2^**	***P*** **-value**	***OR*** **(OR 95% CI)**
How to go to school and come back from school	Active					1.00
	Passive	0.15	0.04	12.61	<0.01	1.16 (1.07–1.25)
How many hours of sleep at night	Adequate					1.00
	Deprivation	0.40	0.08	27.34	<0.01	1.48 (1.28–1.72)
How many hours of video watching every day	<2 h/d					1.00
	≥2 h/d	0.23	0.05	18.59	<0.01	1.25 (1.13–1.39)
Exercising frequency at school	Often					1.00
	Occasional	0.09	0.05	3.00	0.08	1.09 (0.99–1.21)
	Never	0.87	0.07	143.14	<0.01	2.39 (2.07–2.76)
How many hours of exercising at school	≥60 min/d					1.00
	<60 min/d	0.11	0.06	3.48	0.06	1.12 (0.99–1.26)
Exercise after class	Yes					1.00
	No	0.37	0.07	29.27	<0.01	1.45 (1.27–1.66)
How regular they have breakfast	Always					1.00
	Occasional	0.30	0.05	33.00	<0.01	1.35 (1.22–1.50)
	Never	0.64	0.08	61.33	<0.01	1.90 (1.62–2.24)
How often they have snacks	Never					1.00
	Occasional	−0.10	0.06	2.84	0.09	0.91 (0.81–1.02)
	Always	0.24	0.06	14.90	<0.01	1.27 (1.13–1.44)
How often they drink sugared beverages	Never					1.00
	Occasional	0.00	0.05	0.01	0.94	1.00 (0.92–1.10)
	Always	0.32	0.06	26.86	<0.01	1.37 (1.22–1.55)
How picky they are with the food	Never					1.00
	Occasional	0.20	0.05	16.57	<0.01	1.22 (1.11–1.33)
	Always	0.80	0.07	136.05	<0.01	2.24 (1.95–2.56)

*Whether the student has the psychological symptoms (indicated in “yes” and “no”) was the dependent variable and the lifestyle variables were the independent variables and they are: how the students go to and come back from school (active = 1, passive = 2), how many hours of sleep at night (adequate = 1, inadequate = 2), how many hours of using a digital device and watching TV(<2 h/d = 1, ≥2 h/d = 2), how often they exercise (often = 1, occasional = 2, never = 3), how long they exercise (more than 60 min = 1, <60 min = 2), participation in extracurricular exercises (yes = 1, no = 2), how regularly they have breakfast (always = 1, occasionally = 2, never = 3), how often they have snacks (never = 1, occasionally = 2, always = 3), how often they drink sugared drinks (never = 1, drink occasionally = 2, always = 3), how pick they are with the food (never = 1, occasionally = 2, always = 3)*.

*OR, Adjusted Odds Ratio; 95% CI, 95% Confidence interval*.

**Figure 2:**
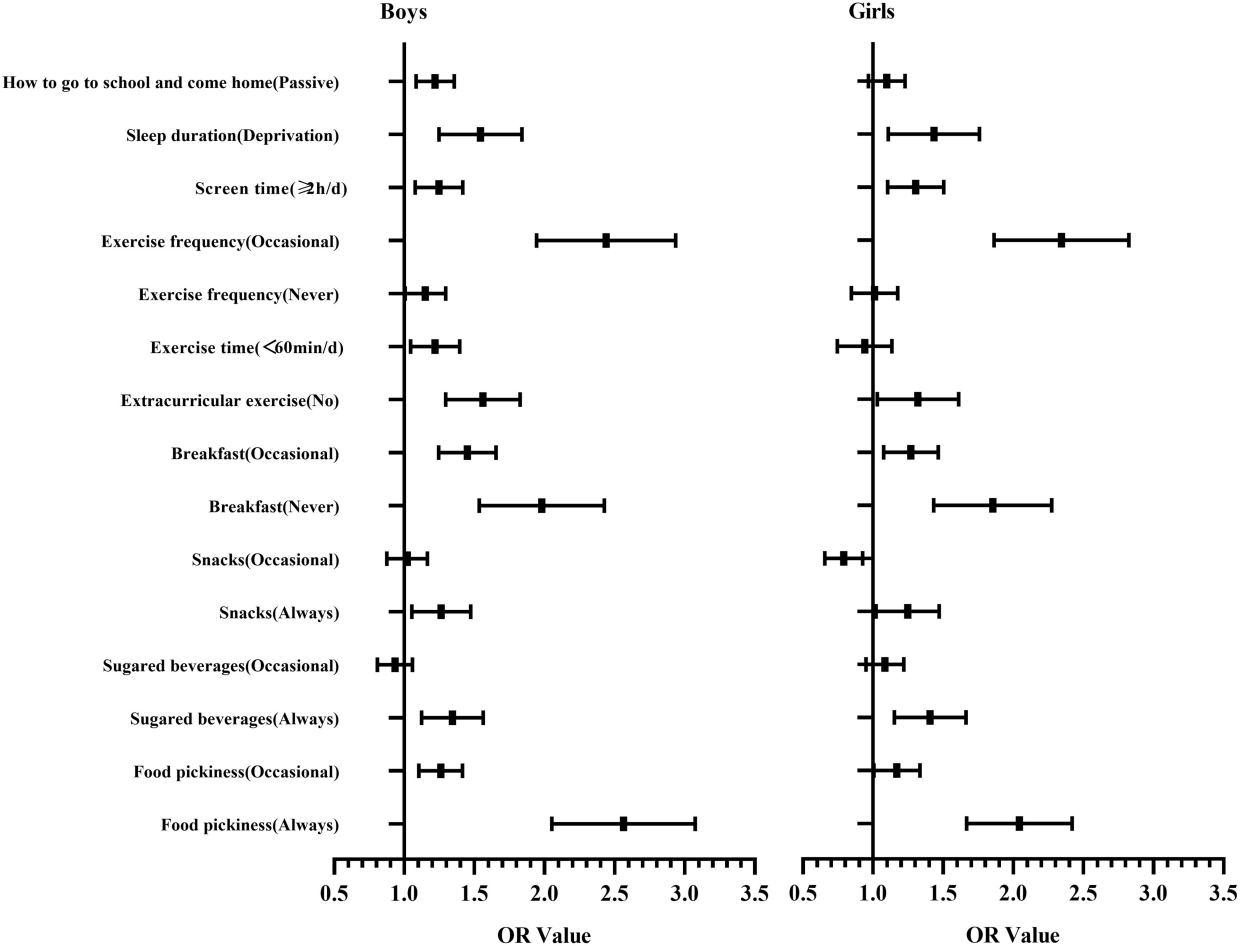
The diagram of the OR value (95% CI) of regression analysis on the correlation between psychological symptoms and lifestyles for boys and girls, respectively.

**Figure 3:**
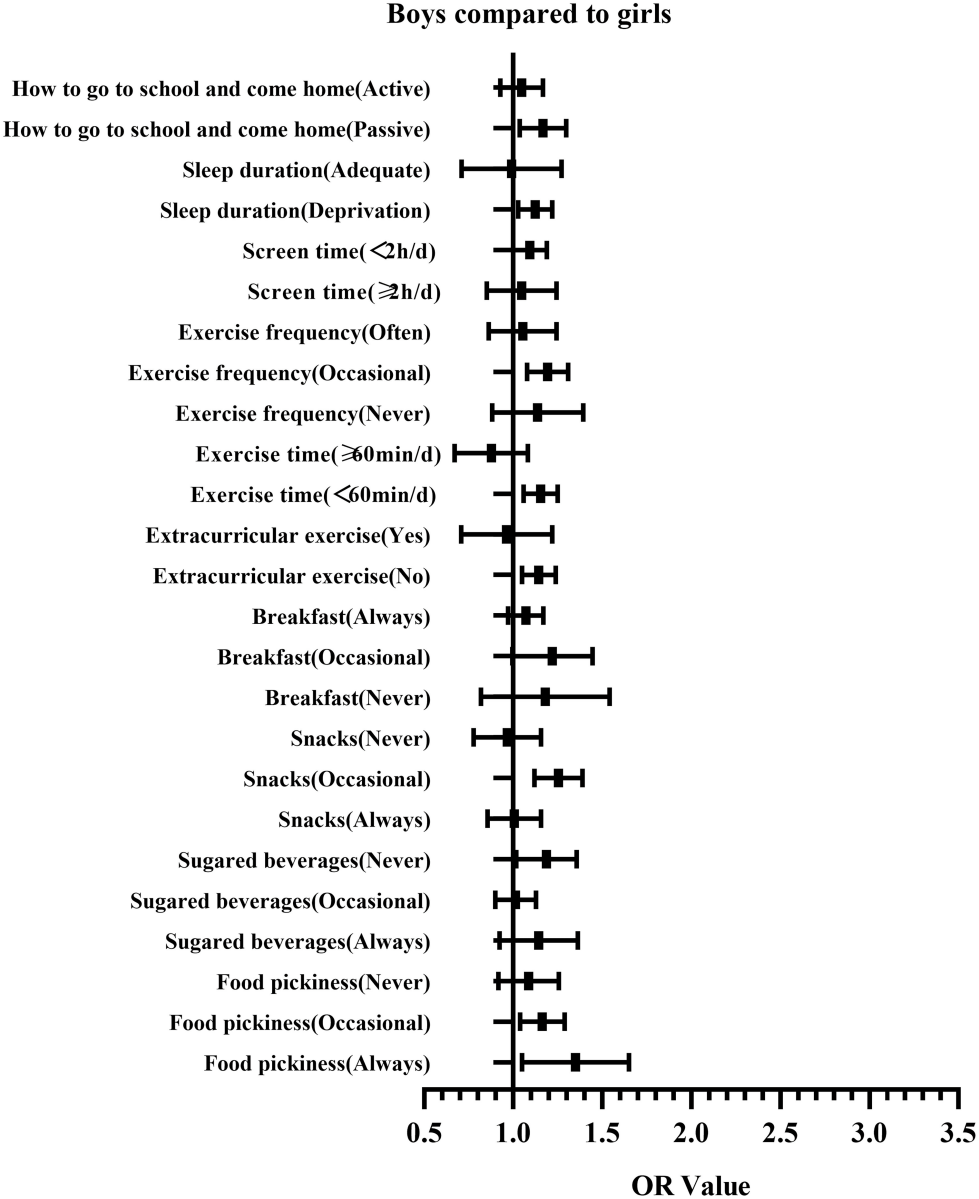
Gender comparison on the correlation between psychological symptoms and lifestyles for Chinese middle school students.

Table 3 and Figure 2 show the regression analysis of the correlation between psychological symptoms and lifestyles. Taking girls as a reference, logistic regression analysis showed that boys have higher OR values, which means they are more easily influenced by lifestyles than girls (Figure 3).

**Table 3:** Regression analysis of the correlation between psychological symptoms and lifestyles.

**Independent variable**		**Boys**		**Girls**		**Boys compared to girls**	
		***P*** **-value**	**OR (95% CI)**	***P*** **-value**	**OR (95% CI)**	***P*** **-value**	**OR (95% CI)**
How to go to school and come back	Active		1.00		1.00	0.48	1.04 (0.93–1.17)
	Passive	<0.01	1.21 (1.09–1.36)	0.13	1.09 (0.97–1.23)	<0.05	1.16 (1.04–1.30)
How many hours of sleep at night	Adequate		1.00		1.00	0.81	0.97 (0.72–1.28)
	Deprivation	<0.01	1.52 (1.26–1.85)	<0.01	1.41 (1.12–1.77)	<0.05	1.12 (1.03–1.22)
How many hours of video watching/using digital device per day	<2 h/d		1.00		1.00	0.05	1.09 (1.00–1.19)
	≥2 h/d	<0.01	1.24 (1.08–1.42)	<0.01	1.29 (1.11–1.51)	0.74	1.03 (0.86–1.25)
Exercising frequency at school	Often		1.00		1.00	0.68	1.04 (0.87–1.25)
	Occasional	<0.01	2.41 (1.96–2.95)	<0.01	2.31 (1.88–2.84)	<0.01	1.19 (1.08–1.31)
	Never	<0.05	1.14 (1.01–1.30)	0.99	1.00 (0.85–1.18)	0.35	1.12 (0.89–1.40)
How many hours of exercising every day	≥60 min/d		1.00		1.00	0.21	0.86 (0.68–1.09)
	<60 min/d	<0.05	1.21 (1.05–1.40)	0.48	0.93 (0.75–1.14)	<0.01	1.15 (1.06–1.25)
Exercise after class	Yes		1.00		1.00	0.67	0.94 (0.72–1.23)
	No	<0.01	1.55 (1.3–1.83)	<0.05	1.30 (1.04–1.62)	<0.01	1.14 (1.05–1.24)
How regular they have breakfast	Always		1.00		1.00	0.18	1.07 (0.97–1.17)
	Occasional	<0.01	1.44 (1.25–1.66)	<0.01	1.26 (1.08–1.47)	0.05	1.21 (1.00–1.45)
	Never	<0.01	1.95 (1.55–2.44)	<0.01	1.82 (1.45–2.29)	0.40	1.14 (0.84–1.56)
How often they have snacks	Never		1.00		1.00	0.65	0.96 (0.78–1.16)
	Occasional	0.88	1.01 (0.88–1.17)	<0.05	0.78 (0.66–0.93)	<0.01	1.25 (1.12–1.39)
	Always	<0.05	1.25 (1.06–1.48)	<0.05	1.23 (1.03–1.48)	0.98	1.00 (0.86–1.16)
How often they have sugared drinks	Never		1.00		1.00	<0.05	1.18 (1.02–1.36)
	Occasional	0.26	0.93 (0.81–1.06)	0.26	1.08 (0.95–1.22)	0.87	1.01 (0.90–1.13)
	Always	<0.01	1.33 (1.13–1.57)	<0.01	1.39 (1.16–1.67)	0.23	1.13 (0.93–1.37)
How picky they are with the food	Never		1.00		1.00	0.37	1.08 (0.92–1.26)
	Occasional	<0.01	1.25 (1.11–1.42)	<0.05	1.16 (1.01–1.34)	<0.05	1.16 (1.04–1.29)
	Always	<0.01	2.53 (2.07–3.09)	<0.01	2.02 (1.68–2.43)	<0.05	1.33 (1.06–1.66)

*OR, Adjusted Odds Ratio; 95% CI, 95% confidence interval*.

## Discussion

The present study used 14,356 data of Chinese middle school students to estimate the correlation between psychological symptoms and lifestyles, finding out that there was a positive correlation between unhealthy lifestyle and the occurrence of psychological symptoms, and boys are more easily influenced by lifestyles than girls.

We found out that the detection rate of psychological symptoms in Chinese middle school students was 21.37%, which is higher than that in studies by Tao et al. (2020) (11.18%) and Wu (2019) (19.65%). Although the same questionnaire was used, the detection rate of psychological symptoms was different because of the different recall levels of the participants. Our study also showed that Chinese boys had a higher detection rate of psychological symptoms than girls, which is consistence with the conclusion of Gu et al. (2017) and Wu (2019). An important reason could be that boys have worse living habits than girls, such as smoking, drinking, and over gaming (Vaezghasemi et al., 2012). The detection rate of the junior middle school students was higher than that of senior middle school students, inconsistent with the research findings of Wen (2020). The reason may be that senior middle school students are better at regulating themselves psychologically (Rao et al., 2019). Besides, the unpredictable environment and unstable minds of junior middle school students added to their susceptibility to the psychological symptoms (Chi et al., 2020).

Students who chose to go to school and come back home in a passive way (take the bus, metro, and automobile) got a higher detection rate of psychological symptoms. Research has confirmed that actively going to school (by foot or riding a bicycle) is beneficial by improving physical activity level, which can in turn influence their psychological development (Weist et al., 2000; Whetten et al., 2006; Kutcher and Wei, 2012; Avitsland et al., 2020). Wang et al. (2020) suggested that the occurrence of psychological symptoms among Chinese teenagers was increasing. The possible reasons may be that their lifestyles have changed, including lower physical levels, and higher sedentary behavior (Rodriguez-Ayllon et al., 2019; Wang et al., 2020).

We also found out that deprivation of sleep and excessively watching videos would lead to a higher risk of them getting psychological symptoms, which is in the agreement with other relevant studies (Cao et al., 2011; Backovic et al., 2013; Riemann, 2018, 2020). It was reported that inadequate sleep or staring at the LED screen too long would keep the brain stimulated or stressed, causing psychological symptoms (Zhang et al., 2020). Similarly, our study suggested that students without exercising were positively correlated with the occurrence of the psychological symptoms, which was consistent with the result of Kandola et al. (2020). Studies have confirmed that psychological symptoms associated with depression and anxiety disorders are increasingly common in obese children and adolescents, and skipping breakfast and drinking sugared beverages beyond normal limits would increase the risk of obesity (Britz et al., 2000; Quek et al., 2017). Therefore, students with these poor-eating habits tend to have psychological symptoms.

Our study showed that, compared with girls, Chinese boys were more easily contract psychological symptoms, and were more susceptible to lifestyle influences. It was reported that unhealthy life would cost physical health and cause bigger risks of getting psychological symptoms (Ekelund et al., 2016; Janssen et al., 2020). A cross-sectional study of Catalonia adolescents showed that girls scored higher on eating behavior and hygiene habits than boys, further supporting the findings of our study (Costa-Tutusaus and Guerra-Balic, 2016). This reminds us that it is time for middle school students, especially boys, to establish healthy lifestyles for better psychological states.

The present study has some advantages. The first advantage of our study lies in the large sample of Chinese middle school students. Second, we found the gender differences in the correlations between the lifestyles and the occurrence of psychological symptoms, which can remind parents and educators to pay attention to boys' mental health.

However, the study was limited in its way. First of all, the study was a cross-sectional investigation and was unable to deduce the causal relations between lifestyles and psychological symptoms and further cohort study is necessary to solve this problem. Second, the present study used a questionnaire to estimate psychological symptoms, which are susceptible to the level of recall, and future studies would be better using the objective measurements method.

## Conclusions

The present study used 14,356 data of Chinese middle school students to estimate the correlation between psychological symptoms and lifestyles, finding out that there was a positive correlation between unhealthy lifestyle and the occurrence of psychological symptoms, and boys are more easily influenced by lifestyles than girls. Targeted measures such as lessons on health lifestyle, and dissemination of health knowledge should be taken for the better mental health of Chinese middle school students.

## Data Availability Statement

The raw data supporting the conclusions of this article will be made available by the authors, without undue reservation.

## Ethics Statement

The studies involving human participants were reviewed and approved by the Ethics Committee of Sports College of Shangrao Normal University (2018R-0219). Written informed consent to participate in this study was provided by the participants' legal guardian/next of kin.

## Author Contributions

JL: conceptualization. JL, MS, and NL: data curation. JL, MS, JQ, and NL: formal analysis and resources. JL and MS: funding acquisition. JL, JZ, MS, YX, and NL: investigation. JL and JZ: methodology and software. MS: project administration. MS, YX, and CT: supervision. JQ and YX: validation. JZ and YX: visualization. JL and CT: writing—original draft and writing—review and editing. All authors have read and agreed to the published version of the manuscript.

## Funding

The research was supported by the 2017 national general project of the 13th Five-Year Plan of National Education Science: Research on Rural School Development (1949–2017) (No. bha170138).

## Conflict of Interest

The authors declare that the research was conducted in the absence of any commercial or financial relationships that could be construed as a potential conflict of interest.

## Publisher's Note

All claims expressed in this article are solely those of the authors and do not necessarily represent those of their affiliated organizations, or those of the publisher, the editors and the reviewers. Any product that may be evaluated in this article, or claim that may be made by its manufacturer, is not guaranteed or endorsed by the publisher.
